# Immunomagnetic Delivery of Adipose-Derived Endothelial Progenitor Cells for the Repair of Renal Ischemia–Reperfusion Injury in a Rat Model

**DOI:** 10.3390/bioengineering10050509

**Published:** 2023-04-24

**Authors:** Di Wu, Jingyu Liu, Changcheng Zhou, Wenjie Ma, Liuhua Zhou, Yuzheng Ge, Ruipeng Jia

**Affiliations:** Department of Urology, Nanjing First Hospital, Nanjing Medical University, Nanjing 210006, China

**Keywords:** magnetic nanoparticles, CD133, EPCs, renal IRI

## Abstract

Renal ischemia–reperfusion injury (IRI) is a significant cause of acute kidney injury (AKI) and usually brings severe public health consequences. Adipose-derived endothelial progenitor cell (AdEPCs) transplantation is beneficial for AKI but suffers from low delivery efficiency. This study was conducted to explore the protective effects of magnetically delivered AdEPCs on the repair of renal IRI. Two types of magnetic delivery methods, namely the endocytosis magnetization (EM) method and the immunomagnetic (IM) method were fabricated using PEG@Fe_3_O_4_ and CD133@Fe_3_O_4_, and their cytotoxicities in AdEPCs were assessed. In the renal IRI rat model, magnetic AdEPCs were injected via the tail vein and a magnet was placed beside the injured kidney for magnetic guidance. The distribution of transplanted AdEPCs, renal function, and tubular damage were evaluated. Our results suggested that CD133@Fe_3_O_4_ had the minimum negative effects on the proliferation, apoptosis, angiogenesis, and migration of AdEPCs compared with PEG@Fe_3_O_4_. Renal magnetic guidance could significantly enhance the transplantation efficiency and the therapeutic outcomes of AdEPCs–PEG@Fe_3_O_4_ and AdEPCs–CD133@Fe_3_O_4_ in the injured kidneys. However, under renal magnetic guidance, AdEPCs–CD133@Fe_3_O_4_ had stronger therapeutic effects than PEG@Fe_3_O_4_ after renal IRI. The immunomagnetic delivery of AdEPCs with CD133@Fe_3_O_4_ could be a promising therapeutic strategy for renal IRI.

## 1. Introduction

As a critical cause of acute kidney injury (AKI), renal ischemia–reperfusion injury (IRI) is a result of a transient reduction or blockage of renal blood flow followed by blood reperfusion [1]. Renal ischemia leads to an imbalance in oxygen supply and demand, and enhances anaerobic metabolism, while subsequent reperfusion is always accompanied by damaged microvascular endothelial cells [2]. Meanwhile, growing evidence illustrates a strong relationship between AKI and subsequent chronic kidney disease, which brings severe public health consequences [3,4,5].

Cell-based therapies have been regarded as a set of promising treatments in biotechnology that promote tissue regeneration and restore the biological functions of damaged tissues [6,7]. The efficacy of cell transplantation depends on several factors, including biological function, the number of transplanted cells, and the retention of the cells at the injury site [8]. To date, the renoprotective roles of endothelial progenitor cells (EPCs) in AKI have been widely reported, possibly by increasing neovascularization, suppressing inflammation, and secreting extracellular vesicles to deliver specific microRNAs (miRNAs) [9,10,11,12,13,14]. The vast majority of EPCs used in current studies are derived from bone marrow or peripheral blood, which is time-consuming and inconvenient [15]. Previous studies of our group successfully identified and cultured adipose-derived endothelial progenitor cells (AdEPCs), which provided a new and convenient source for EPC transplantation [16,17]. Moreover, the extracted primary AdEPCs highly expressed CD133, a stem cell biomarker [17]. Previous studies have established that CD133^+^ EPCs represent a stronger promotion of angiogenesis and have better therapeutic potential [18,19,20,21,22,23,24]. Notably, CD133^+^ EPC transplantation in critical limb ischemia was reported in several clinical trials and yielded encouraging therapeutic outcomes [25,26]. CD133^+^ AdEPCs may be a promising therapeutical cell source for renal IRI. 

In the studies of cell-based therapy in AKI, only about 3% of intravenously transplanted cells reached the kidney and engrafted [27]. In emerging nanomedicine, magnetic nanoparticles (MNPs), as a medical tool for cells, drugs, growth factors, and gene delivery, have been extensively studied [28]. After systematically transplanting magnetized cells under magnetic guidance, high delivery efficiency and a better therapeutic outcome could be observed [29,30]. Based on different mechanisms, there are two different methods to magnetize stem cells: the endocytosis magnetization (EM) method and the immunomagnetic (IM) method. EM is usually performed by cultivating cells with a certain concentration of MNPs for a few hours, and the cells uptake the MNPs by passive endocytosis [8,29,31,32]. However, this type of magnetization is time-consuming and less cell-specific. More importantly, excessive intracellular uptake of iron oxide may have a toxic impact on the biological properties of treated cells, including cell membrane damage, protein denaturation, and genotoxicity [33,34,35,36]. These potential toxicities may be avoided by IM. 

Herein, the present study aimed to identify a magnetic non-invasive AdEPCs transplantation strategy for renal IRI repair. To the best of our knowledge, there are no relevant studies on the magnetic delivery of AdEPCs for renoprotection. Based on EM and IM, we fabricated two types of MNPs, namely PEG@Fe_3_O_4_, and CD133@Fe_3_O_4_, to magnetize AdEPCs and further explored their effects on AdEPCs transplantation-mediated therapeutic potential for renal IRI.

## 2. Materials and Methods

### 2.1. Preparation and Characterization of MNPs 

Oleic acid-coated Fe_3_O_4_ (Fe_3_O_4_@OA) was synthesized by the thermal decomposition method according to previous studies [37,38]. For the fabrication of PEG@Fe_3_O_4_, DSPE-PEG2000-COOH and DSPE-PEG2000 powder were mixed in chloroform and reacted with Fe_3_O_4_@OA in a rotary evaporator (BUCHI Rotavapor™ R-300, Fisher Scientific, Waltham, MA, USA) at 70 °C to form a hydrophilic shell on the Fe_3_O_4_@OA core. The synthesized PEG@Fe_3_O_4_ has a 20 nm Fe_3_O_4_@OA core and a hydrophilic shell of DSPE-PEG2000 with -COOH terminus.

**Figure 1:**
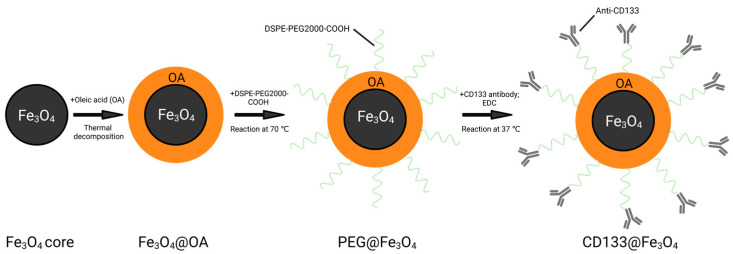
Schematic representation of the fabrication of PEG@Fe_3_O_4_ and CD133@Fe_3_O_4_. Created with BioRender.com.

For the fabrication of CD133@Fe_3_O_4_, besides the synthesized PEG@Fe_3_O_4_, CD133 antibody (PA5-38014, Thermo Scientific, Shanghai, China), and 1-(3-Dimethylaminopropyl)-3-ethylcarbodiimide hydrochloride (EDC; Sigma-Aldrich, Shanghai, China) were used. In the presence of carbodiimide analogues, the carboxyl group reacts with the amino group to form a couple of amide bonds between the CD133 antibodies and the surface of PEG@Fe_3_O_4_. Briefly, the synthesized 20 nm PEG@Fe_3_O_4_ (1 mg/mL) was filtered in a 20 nm magnetic sorter column, washed once with MES (15 mM, pH = 5.5), and resuspended using ultrapure water. EDC (10 mg/mL) was added to the purified PEG@Fe_3_O_4_ to activate the -COOH groups of PEG@Fe_3_O_4_ with vortex, after which, CD133 antibody (1 mg/mL) was added and mixed. The reaction was continued for 20 h under vibration at 37 °C. Afterward, the reaction solution was collected and purified by a 20 nm magnetic sorting column (Figure 1). To determine whether AdEPCs are specifically bound to CD133@Fe_3_O_4_, we used the isotype control of CD133 antibody (Rabbit IgG Isotype Control, 31235, Thermo Scientific, Shanghai, China) to fabricate Iso@Fe_3_O_4_.

The antibody coupling rate of CD133@Fe_3_O_4_ was determined by measuring the full range UV absorption spectra before and after the CD133 antibody reaction. The hydrodynamic sizes and stability of CD133@Fe_3_O_4_ and PEG@Fe_3_O_4_ were examined by dynamic light scattering (DLS; Zetasizer Lab, Malvern Panalytical Ltd., Malvern, UK) measurements and zeta potential measurements (Zetasizer Lab, Malvern Panalytical Ltd., Malvern, UK), respectively. Their magnetic properties were assessed by a vibrating sample magnetometer (VSM; Vibration magnetometer 7407, Lake Shore Cryotronics, Westerville, OH, USA). Finally, the morphological structures of CD133@Fe_3_O_4_ and PEG@Fe_3_O_4_ were observed by a transmission electron microscope (TEM; HT7800, Hitachi, Tokyo, Japan) operating at 120 kV.

### 2.2. Animals and AdEPCs Isolation

The Ethics Committee for the Use of Experimental Animals at Nanjing First Hospital, Nanjing Medical University, authorized all animal experimentation protocols. Male Sprague Dawley rats weighing 200–300 g were handled in complete conformity with the National Institutes of Health’s Guidelines for the Care and Use of Laboratory Animals.

The isolation and culture of AdEPCs were according to the protocols published by our group with minor modifications [16]. Briefly, rats’ adipose tissues from the epididymis were collected through a median incision in the lower abdomen. Adipose tissues were cut into small pieces and placed in 0.1% type I collagenase for digestion at 37 °C with gentle shaking for 30 min. Primary stromal vascular fraction (SVF) cells were obtained by centrifugation, lysis of erythrocytes, and repeated washing. Finally, cells were resuspended in endothelial cell growth medium-2 (EGM-2; Lonza, Basel, Switzerland) containing 5% fetal bovine serum (FBS; 10099141C, Thermo Scientific, Shanghai, China), seeded in a 25 cm^2^ cell culture flask, and incubated at 37 °C in a 5% CO_2_ incubator (Heraeus BB 150 CO_2_ incubator, Thermo Scientific, Shanghai, China).

By the fifth day of culture, a mixture of long spindle-shaped mesenchymal stem cells and short spindle-shaped endothelial progenitor cells could be observed. Since endothelial progenitor cells have a strong wall-adhesive ability, the difference in the ability of different cells to respond to trypsin digestion was used for primary cell purification. The purified cells were cultured with EGM-2 supplemented with 10% FBS in the previous culture flask.

### 2.3. Identification of AdEPCs

Dil-acetylated low-density lipoprotein (Dil-Ac-LDL; H7970, Solarbio, Beijing, China) uptake and fluorescein isothiocyanate-labeled Ulex europaeus agglutinin-1 (FITC-UEA-1; L9006, Sigma-Aldrich, Shanghai, China) binding assays, immunofluorescence staining (against CD31, CD34, CD133, and VEGFR2) and flow cytometry was used to identify the subcultured AdEPCs. For all experiments, AdEPCs were used in passages 2–4. For Dil-Ac-LDL uptake and the FITC-UEA-1 binding assay, briefly, after incubation with 10 µg/mL Dil-Ac-LDL at 37 °C for 4 h, the subcultured cells were washed with PBS (pH = 7.4) thrice and then fixed with 4% prechilled paraformaldehyde for 20 min. After fixation and washing, cells were incubated with 10 µg/mL FITC-UEA-1 for 1 h at room temperature, and, finally, the nuclei were stained using 4,6-diamidino-2-phenylin-dole (DAPI; P0131, Beyotime, Shanghai, China). For immunofluorescence staining, cells were incubated overnight at 4 °C with a set of primary antibodies including anti-CD31 (ab222783, Abcam, Cambridge, UK), anti-CD34 (ab81289, Abcam, Cambridge, UK), anti-CD133 (ab19898, Abcam, Cambridge, UK), and anti-VEGFR2 (ab2349, Abcam, Cambridge, UK). After washing three times with PBS and incubating with Alexa Fluor 488 or 555 (ab150077, ab150078, Abcam, Cambridge, UK) for 1 h at room temperature, the nuclei were finally stained with DAPI. The treated cells were observed using a fluorescent microscope (Ti-S, Nikon, Tokyo, Japan). For flow cytometry, AdEPCs.

Identification was performed as described in our previous protocol. Briefly, a set of fluorescent antibodies—anti-CD133-FITC (orb15325, Biorbyt, Wuhan, China), anti-CD34-PE (bs-0646R-PE, Bioss, Boston, MA, USA), anti-CD31-APC (bs-0195R-APC, Bioss, Boston, USA), anti-VEGFR2-FITC (bs-10412R-FITC, Bioss, Boston, USA), anti-CD45-PE (bs-0522R-PE, Bioss, Boston, MA, USA), and anti-CD14-FITC (bs-1192R-FITC, Bioss, Boston, MA, USA)—were used to determine the surface marker expression of AdEPCs. An isotype-matched IgG was used as the negative control for each primary antibody.

### 2.4. Magnetization of AdEPCs with MNPs

For loading with PEG@Fe_3_O_4_, cells were seeded in a six-well plate and incubated with different concentrations of PEG@Fe_3_O_4_ (0 µg/mL, 25 µg/mL, 50 µg/mL, and 100 µg/mL) in 2 mL EGM-2 supplemented with 10% FBS overnight. After washing thrice with PBS to remove excess nanoparticles, cells were collected for subsequent experiments.

For labeling with CD133@Fe_3_O_4_ or Iso@Fe_3_O_4_, after growing to 80% fusion in a six-well plate, cells were collected in a 15 mL centrifuge tube by centrifugation using 0.25% trypsin/0.038% ethylene diamine tetraacetic acid (EDTA; Thermo Scientific, Shanghai, China) and incubated with different concentrations of CD133@Fe_3_O_4_ (0 µg/mL, 25 µg/mL, 50 µg/mL, and 100 µg/mL) and Iso@Fe_3_O_4_ (50 µg/mL) in 2 mL complete EGM-2 medium at 37 °C with gentle shaking for 1 h.

### 2.5. Examination of AdEPCs Magnetization

A Prussian blue staining kit (G1422, Solarbio, Beijing, China) was used to localize iron particles of AdEPCs–PEG@Fe_3_O_4_ and AdEPCs–CD133@Fe_3_O_4_ under the bright field. The experimental procedure was performed according to the manufacturer’s instructions. 

For further localization of AdEPCs–CD133@Fe_3_O_4_ with immunofluorescence staining, the AdEPCs labeled with CD133@Fe_3_O_4_ (50 µg/mL) were fixed with 4% paraformaldehyde for 15 min and blocked at room temperature for 1 h. The secondary antibody (red) was goat anti-rabbit IgG (H+L) (ab6702, Abcam, Cambridge, UK) and was used at 2 µg/mL for 1 h to stain CD133@Fe_3_O_4_. Amanita phalloides (green) P5282 (Sigma-Aldrich, Shanghai, China) was used at 1 µg/mL for 30 min to stain cellular actin. DAPI (blue) was used for 5 min to stain the cell nuclei.

The TEM images of AdEPCs–PEG@Fe_3_O_4_ and AdEPCs–CD133@Fe_3_O_4_ were obtained with a Hitachi HT7800 electron microscope operating at 120 kV.

### 2.6. Cell Apoptosis Assay

AdEPCs (5 × 10^5^ cells per group) were incubated with different concentrations of CD133@Fe_3_O_4_ or PEG@Fe_3_O_4_ overnight. The magnetized cells were washed twice with PBS and collected. An apoptosis detection kit (KGA101, KeyGEN BioTECH, Nanjing, China) was used. Briefly, the cells were suspended with 500 µL binding buffer and incubated with 5 µL annexin V-EGFP and propidium iodide for 15 min at room temperature protected from light. A flow cytometer (C40323, Beckman Coulter, Indianapolis, IN, USA) was used to measure the treated cells, and then flow cytometer software version V10 (Beckman Coulter, Indianapolis, IN, USA) was used for data analysis.

### 2.7. Cell Proliferation Assay

The growth of the magnetized AdEPCs was evaluated using Cell Counting Kit-8 (CCK-8) (C0037, Beyotime, Shanghai, China) assays. Briefly, the treated AdEPCs were seeded in 96-well plates (2000 cells/well) and incubated for 24 h or 5 consecutive days. The optical density (OD) was examined at the absorbance of 450 nm using a microplate reader (Infinite F500, Tecan, Männedorf, Switzerland). 

For the growth of HUVECs co-cultured with AdEPCs, Ki-67 immunofluorescent staining was used. The cells were seeded in 12-well plates (10^5^ cells/well). After fixation and blocking, cells were incubated with primary antibody Ki-67 (ab15580, Abcam, England) overnight at 4 °C. Then, a secondary antibody (green) (ab150077, Abcam, Cambridge, England) and DAPI (blue) were used.

### 2.8. Detection of ROS Levels

The reactive oxygen species (ROS) levels of AdEPCs–PEG@Fe_3_O_4_ and AdEPCs–CD133@Fe_3_O_4_ were detected using a ROS detection kit (S0033S, Beyotime, Shanghai, China) according to the manufacturer’s instructions. Briefly, 2 × 10^5^ AdEPCs were seeded in a six-well plate. When the cell fusion grew to about 80%, 50 µg/mL of AdEPCs–PEG@Fe_3_O_4_ and AdEPCs–CD133@Fe_3_O_4_ were added, respectively, and incubated overnight. After that, 10 µM of DCFH-DA was added and incubated for a further 20 min at 37 °C. Then, the cells were washed thrice with PBS and detected with a flow cytometer (C40323, Beckman Coulter, Indianapolis, IN, USA).

### 2.9. Migration Assay

The effects of PEG@Fe_3_O_4_ or CD133@Fe_3_O_4_ on AdEPCs were assessed using scratch assays and transwell assays. For scratch assays, cells were seeded in a six-well plate, and after cell adherence the serum-free medium was replaced to stop cell proliferation. A uniform scratch of the cell monolayer was created by a 200 µL pipette tip. The closing areas of the identical scratch area of each group at 0, 6, 12, and 18 h were observed under an inverted microscope (IX51, Olympus, Tokyo, Japan) and measured with ImageJ software Version 1.53t (Rasband, W.S., ImageJ, U. S. National Institutes of Health, Bethesda, MD, USA). The migration rate was calculated using the following formula:

Migration Rate (%) = (A_0_ − A_t_)/A_0_ × 100, (1)

where A_0_ represents the initial scratch area, and A_t_ represents the remaining scratch area at the time of measurement. 

Transwell assays were performed for six groups: AdEPCs, and AdEPCs–PEG@Fe_3_O_4_ and AdEPCs–CD133@Fe_3_O_4_ both treated with or without an external magnetic field. Briefly, the cells suspended in 100 µL serum-free EGM-2 medium were seeded in the upper chamber of a 24-well transwell (diameter of pores: 8 µm, Corning, NY, USA) while 500 µL EGM-2 medium with 10% FBS was added to the lower compartment. After incubation for 24 h and fixation for 15 min, the cells that migrated to the lower surface were stained with 0.1% crystal violet. The images of 6 nonoverlapping random fields from each well were obtained using a microscope (Ti-S, Nikon, Tokyo, Japan). 

### 2.10. Tube Formation Assay

Matrigel (10 µL/well) (354248, Corning, NY, USA) was added into a pre-cooled μ-slide angiogenesis plate (81506, Ibidi, Gräfelfing, Germany) and solidified at 37 °C for 30 min. The cells (10^4^/well) suspended in 50 µL serum-free EGM-2 medium were seeded onto the former matrigel-coated plates. After incubation for 6 h, the tube formation was quantified in total tube length and the number of branch points using phase-contrast microscopy (Ti-S, Nikon, Tokyo, Japan).

### 2.11. Rat Renal IRI Model 

After rats were successfully anesthetized with sodium pentobarbital (50 mg/kg; IP), the right kidney was removed through a 2 cm incision on the back, and the wound was properly sutured. All intravenously injected cells with or without magnetization were additionally labeled with the CellTracker™ CM-Dil (C7001, Molecular Probes, Thermo Fisher Scientific, Waltham, MA, USA) and Dil-C18(5)-DS (D12730, Molecular Probes, USA). Briefly, collected cells were incubated with 2 µg/mL of CM-Dil and Dil-C18(5)-DS at 37 °C for 5 min, then incubated at 4 °C for 20 min, and finally washed thrice with PBS.

Two weeks after the right nephrectomy, rats were randomly divided into 6 groups: (a) sham group—rats were subjected to separating the left renal artery without clamping; (b) IRI group—as previously described [39], rats were subjected to clamping the left renal arteries with a non-traumatic vascular clamp for 40 min (IR procedure) and 200 µL PBS was injected into the tail vein; (c) AdEPCs group—rats were subjected to the IR procedure and injected with 2 × 10^6^ AdEPCs dispersed in 200 µL PBS via the tail vein; (d) AdEPCs–CD133@Fe_3_O_4_ group—rats were subjected to the IR procedure and injected with 2 × 10^6^ AdEPCs–CD133@Fe_3_O_4_ via the tail vein; (e) AdEPCs–PEG@Fe_3_O_4_+M group—rats were subjected to the IR procedure and injected with 2 × 10^6^ AdEPCs–PEG@Fe_3_O_4_ via the tail vein, and a magnet was placed next to the left kidney for 30 min; (f) AdEPCs–CD133@Fe_3_O_4_+M group—rats were subjected to the IR procedure and injected with 2 × 10^6^ AdEPCs–CD133@Fe_3_O_4_ via the tail vein, and a magnet was placed next to the left kidney for 30 min; (g) AdEPCs–Iso@Fe_3_O_4_+M group—rats were subjected to the IR procedure and injected with 2 × 10^6^ AdEPCs–Iso@Fe_3_O_4_ via the tail vein, and a magnet was placed next to the left kidney for 30 min. For each experimental procedure, 12 rats per group were used. Moreover, 6 rats from each group were sampled and executed at 24 and 72 h after reperfusion, respectively.

### 2.12. Cell Tracking

An IVIS Spectrum in vivo imaging system (124262, PerkinElmer, Waltham, MA, USA) and fluorescence microscope (Ti-S, Nikon, Tokyo, Japan) were used successively for tracking the distribution of AdEPCs in major organs of IRI rats. Briefly, the heart, lung, liver, spleen, and kidney specimens of treated rats were harvested after 24 h of reperfusion, followed by detection with the IVIS Spectrum in vivo imaging system and analysis with Living Image 4.5 software (Caliper Life Sciences, Hopkinton, MA, USA). Afterward, the tissues were frozen within the OCT compound (Tissue-Tek, Sakura Finetek, Osaka, Japan) and then cut into 5 µm cryosections using a frozen sectioning machine. After rewarming, fixation and closure, the sections were incubated with anti-CD133 (ab19898, Abcam, Cambridge, UK) and DAPI (P0131, Beyotime, Shanghai, China). The CM-Dil (red) and CD133 (green) positive cells were detected with a fluorescence microscope (Ti-S, Nikon, Tokyo, Japan). The area of CM-Dil positive cells was evaluated with ImageJ software (Rasband, W.S., ImageJ, U. S. National Institutes of Health, Bethesda, MD, USA).

### 2.13. Renal Function Analysis

The renal function of different groups was characterized by serum renal injury marker levels, including blood urine nitrogen (BUN) and serum creatinine (SCr). After 24 and 72 h of reperfusion, 5 mL of blood was collected from the inferior vena cava of each group of rats and centrifuged at 2000 rpm for 20 min to extract the supernatants. Clinically automated analysis methods (7180 Clinical Analyzer, Hitachi, Tokyo, Japan) were used to measure the SCr and BUN of the extracted supernatants.

### 2.14. Histological and Immunohistochemical Examination

Tubular damage of IRI kidneys was assessed by hematoxylin and eosin (H&E) staining. The kidney samples were collected from each group after 24 and 72 h of reperfusion and made into 5 µm sections as described previously [40]. After H&E staining, histopathological scoring was evaluated in a blinded format by two experienced pathologists. Based on the calculation of the percentage of damaged tubules (six consecutive fields per section from at least 6 rats per group), the degree of kidney tubular injury was scored using the histological score of the kidney, ranging from Grade 0 to 5 (0: 0%; 1: <10%; 2: 10–25%; 3: 26–45%; 4: 46–75% 5: >75%). 

The effects of MNPs and renal magnetic guidance on cell proliferation, apoptosis, and small vascular density were assessed 72 h after reperfusion. Briefly, after deparaffinized, rehydrating, antigen retrieval, and blocking of the kidney sections, the anti-proliferating cell nuclear antigen (anti-PCNA) antibody (ab92552, Abcam, Cambridge, England) was used for the examination of renal cell proliferation. According to the manufacturer’s instructions, a terminal transferase-mediated deoxyuridine triphosphate nick-end-labeling (TUNEL) assay (Roche, Basel, Switzerland) was performed to determine the apoptosis of renal cells. Microvessel density (MVD) was quantified by the number of microvessels per high-power field after staining with the anti-CD34 antibody (ab81289, Abcam, Cambridge, UK). 

### 2.15. HUVECs and AdEPCs Co-Culture Model

Human umbilical vein endothelial cells (HUVECs) were seeded in a 12-well plate with 1.5 mL complete Dulbecco’s Modified Eagle Medium (DMEM; Thermo Scientific, Shanghai, China). For the hypoxia–reoxygenation (H/R) procedure, briefly, HUVECs were cultured in a 37 °C incubator under hypoxic conditions (94% N_2_, 5% CO_2_, and 1% O_2_) for 8 h and then reoxygenated by incubating for another 8 h under standard culture conditions.

A magnetic sorting column was used for sorting CD133^+^ AdEPCs from AdEPCs–CD133@Fe_3_O_4_. After flowing through the sorting column, CD133^+^ and CD133^−^ AdEPCs were collected and analyzed by flow cytometry (C40323, Beckman Coulter, Indianapolis, IN, USA) to determine the percentage of CD133^+^ cells in the positively selected fraction as well as CD133^−^ cells in the negatively selected fraction. The separated CD133^−^ and CD133^+^AdEPCs (10^5^ cells/well) were cultured in the 12-well transwell chambers (diameter of pores: 0.4 µm; Corning, NY, USA) with complete DMEM for 2 days and then the medium was renewed. Three different 12-well transwell chambers containing DMEM alone, CD133^−^ AdEPCs with DMEM, and CD133^+^ AdEPCs with DMEM were inserted in the 12-well-plate seeded H/R-treated HUVECs under standard culture conditions. After undergoing incubation for 24 h, the treated HUVECs were collected and assessed using tube formation assays, Ki-67 immunofluorescent staining assays, and cell apoptosis assays.

### 2.16. Statistical Consideration

All data were expressed as mean ± standard deviation. Comparisons between two groups were assessed by independent sample *t*-tests, while comparisons between multiple groups were evaluated by one-way analysis of variance (ANOVA) with the post hoc Tukey test. The value of *p* < 0.05 was considered to have statistical significance.

## 3. Results

### 3.1. Isolation and Identification of AdEPCs

**Figure 2:**
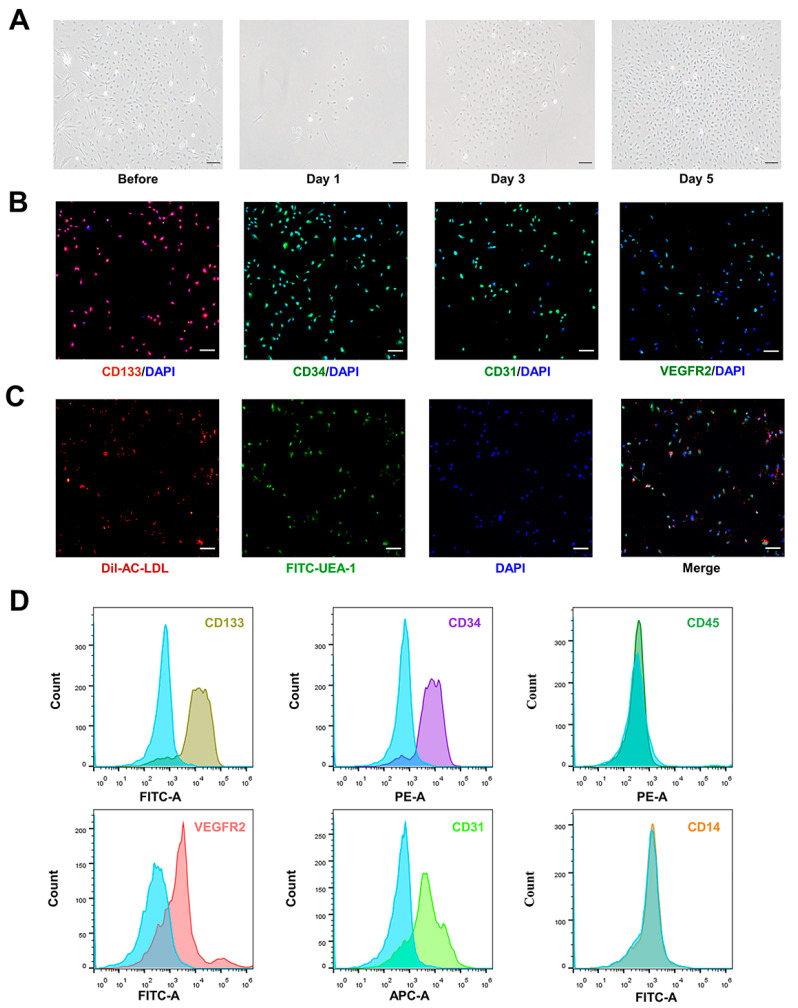
Isolation and identification of AdEPCs. (**A**) Mixed growth of AdEPCs and other cell types before trypsin differential digestion. Typical cobblestone-like cells emerged after trypsin differential digestion and culture for 5 days. (**B**) Representative immunofluorescence images of ad-EPCs specific markers and DNA staining. (**C**,**D**) Dil-Ac-LDL uptake, FITC-UEA-1 binding assay, and flow cytometry further indicated that the cultured cells were EPCs. Scale bar = 100 μm.

After primary SVF inoculation in culture flasks, cell growth was fused to 80% in approximately 5 days. Using the different cell responses to trypsin digestion, AdEPCs were purified, and they exhibited a strong proliferation capacity (Figure 2A). To identify the extracted primary AdEPCs further, we took the second passaged cells for immunofluorescence staining of CD133, VEGFR2, CD31, and CD34 (Figure 2B). Under fluorescence microscopy, positive expression of these markers could be observed in the vast majority of cells. Flow cytometry further supported these results and suggested that AdEPCs barely express CD14 and CD45 (Figure 2D). Additionally, we confirmed the ability of AdEPCs to uptake Dil-Ac-LDL and bind FITC-UEA-1 through immunofluorescence staining (Figure 2C). All these results indicated that we successfully isolated AdEPCs with high expression of CD133.

### 3.2. Characterization of PEG@Fe_3_O_4_ and CD133@Fe_3_O_4_

**Figure 3:**
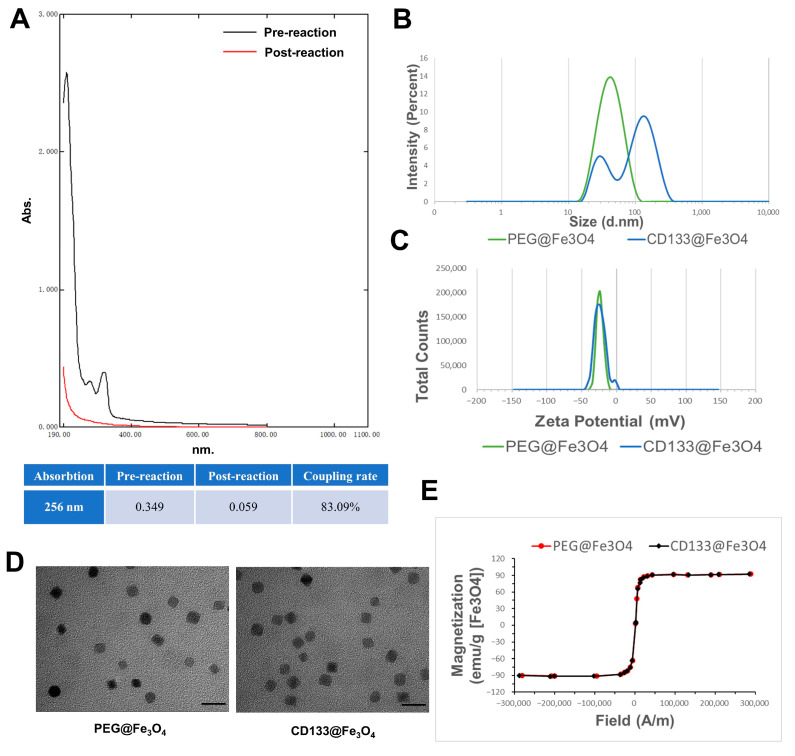
Characterization of PEG@Fe_3_O_4_ and CD133@Fe_3_O_4_. (**A**) The image of full wavelength absorption spectra and CD133 antibody coupling rate calculation before and after CD133 antibody reaction. (**B**) Results of PEG@Fe_3_O_4_ and CD133@Fe_3_O_4_ hydrodynamic size detection. (**C**) Results of PEG@Fe_3_O_4_ and CD133@Fe_3_O_4_ zeta potential detection. (**D**) Representative TEM images of PEG@Fe_3_O_4_ and CD133@Fe_3_O_4_. Scale bar = 40 nm. (**E**) The hysteresis loop diagram of PEG@Fe_3_O_4_ and CD133@Fe_3_O_4_.

As shown in Figure 3, we characterized the physicochemical properties of the synthesized PEG@Fe_3_O_4_ for EM and CD133@Fe_3_O_4_ used for IM. By detecting the full-range UV absorption spectra of the solution before and after the CD133 antibody reaction, the CD133 antibody coupling rate of CD133@Fe_3_O_4_ was larger than 83.09% (Figure 3A). The average zeta potential and hydrodynamic size of PEG@Fe_3_O_4_ were about 23.70 ± 0.89 mV and 44.99 ± 1.24 nm, respectively. After conjugating with CD133, these physicochemical properties of CD133@Fe_3_O_4_ changed to 22.79 ± 0.71 mv and 109.71 ± 1.57 nm, respectively, indicating that both PEG@Fe_3_O_4_ and CD133@Fe_3_O_4_ had good physical stability and were permitted for biomedical applications (Figure 3B,C) [41]. TEM images showed that their particle diameters were all around 20 nm and there was no difference found in their morphologies (Figure 3D). Moreover, the hysteresis loops indicated a relatively high saturation magnetization of 92 emu/g Fe_3_O_4_ (Figure 2E).

### 3.3. AdEPCs Magnetization with PEG@Fe_3_O_4_ and CD133@Fe_3_O_4_

**Figure 4:**
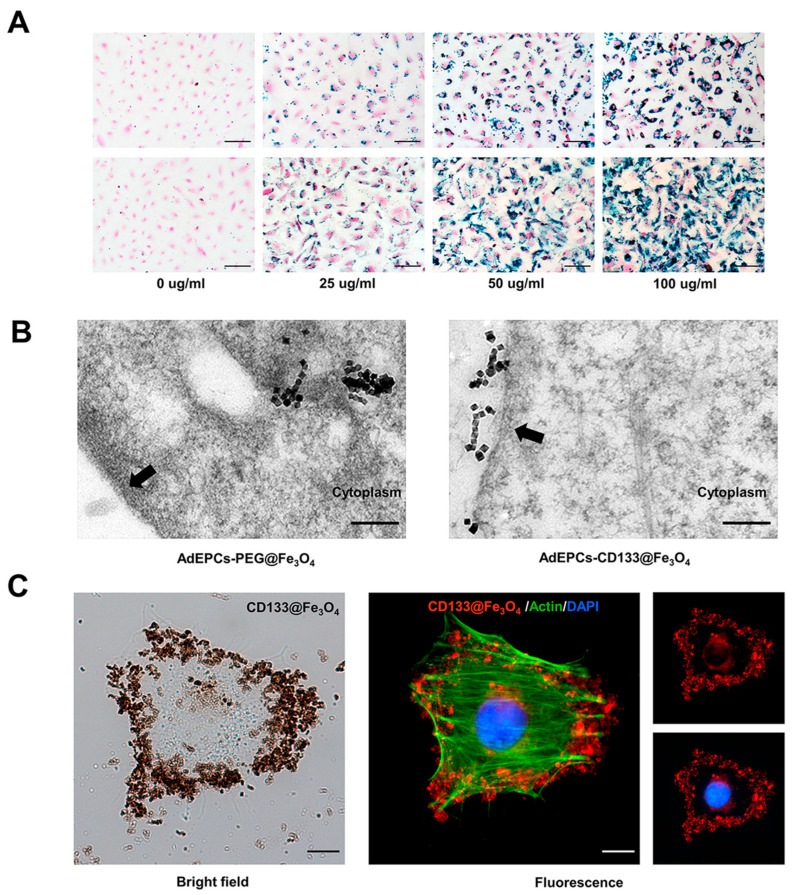
AdEPCs were magnetized with PEG@Fe_3_O_4_ and CD133@Fe_3_O_4_. (**A**) Representative images of AdEPCs incubated with different concentrations of PEG@Fe_3_O_4_ (above) and CD133@Fe_3_O_4_ (below) after Prussian blue staining. Scale bar = 100 μm. (**B**) TEM images show PEG@Fe_3_O_4_ in the cytoplasm and CD133@Fe_3_O_4_ on the membrane (black arrow) of AdEPCs. Scale bar = 50 nm. (**C**) Representative bright field images and immunofluorescence images of AdEPCs labeled with CD133@Fe_3_O_4_ using a fluorescent secondary antibody (red), Phalloidin-iFluor (green), and DAPI (blue). Scale bar = 10 μm.

To observe the magnetization using EM or IM at the cellular level, Prussian blue staining, immunofluorescence staining, and TEM were adopted. After Prussian blue staining, both PEG@Fe_3_O_4_ and CD133@Fe_3_O_4_ turned blue, with PEG@Fe_3_O_4_ located inside the cell membrane while CD133@Fe_3_O_4_ was located on the cell membrane. Moreover, the density of MNPs was closely related to their incubation concentration (Figure 4A). The ultrastructure of AdEPCs–PEG@Fe_3_O_4_ and AdEPCs–CD133@Fe_3_O_4_ are shown in Figure 4B, and the TEM images further demonstrated that these two different methods of cell magnetization were feasible. Moreover, immunofluorescence staining of AdEPCs–CD133@Fe_3_O_4_ (CD133@Fe_3_O_4_ at 50 µg/mL) showed that numerous CD133@Fe_3_O_4_ were bound to the membrane of AdEPCs (Figure 4C). 

### 3.4. The Influences of PEG@Fe_3_O_4_ and CD133@Fe_3_O_4_ on the Function of AdEPCs

**Figure 5:**
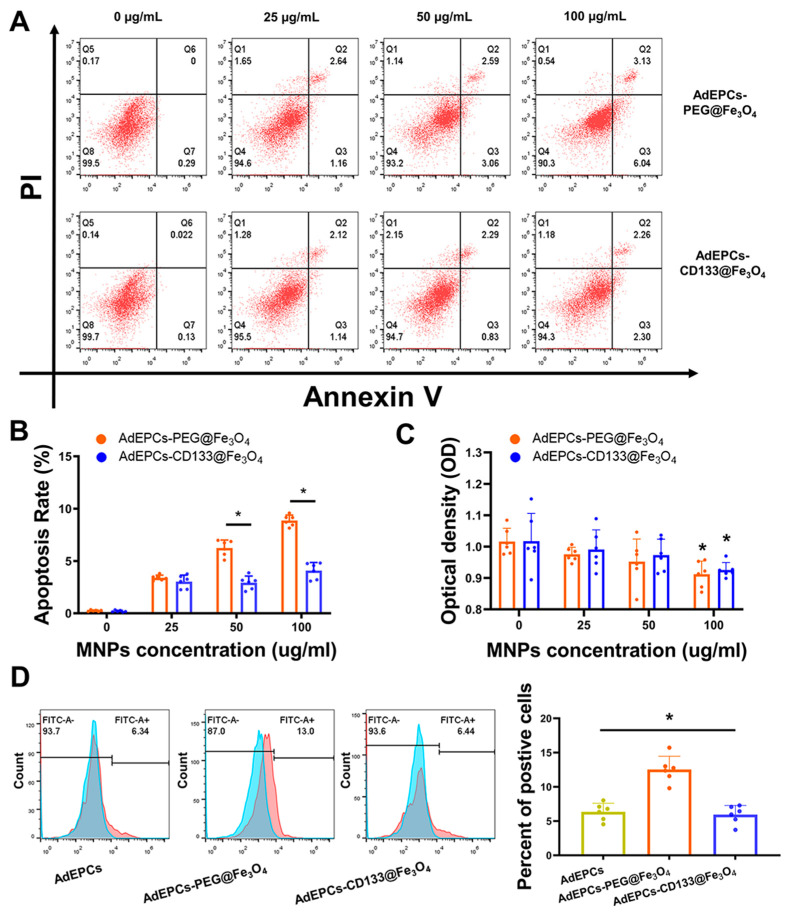
The influences of different concentrations of PEG@Fe_3_O_4_ and CD133@Fe_3_O_4_ on the function of AdEPCs. (**A**,**B**) The apoptosis rate of AdEPCs incubated with different concentrations of PEG@Fe_3_O_4_ and CD133@Fe_3_O_4_ for 24 h. PI, propidium iodide. * *p* < 0.05. (**C**) The proliferation of AdEPCs grown in the medium containing different concentrations of PEG@Fe_3_O_4_ and CD133@Fe_3_O_4_ for 24 h was detected with a cell counting kit. * *p* < 0.05 vs. 0 μg/mL. (**D**) The determination of AdEPCs ROS production after incubating with PEG@Fe_3_O_4_ and CD133@Fe_3_O_4_ at 50 μg/mL for 24 h. * *p* < 0.05.

Apoptosis assays were performed to assess the cytotoxicity and further confirmed the optimal labeling concentration of PEG@Fe_3_O_4_ and CD133@Fe_3_O_4_ used for AdEPCs magnetization. The apoptosis rate of AdEPCs was positively correlated with the concentration of PEG@Fe_3_O_4_ or CD133@Fe_3_O_4_. Moreover, at the labeling concentrations of 50 µg/mL and 100 µg/mL, PEG@Fe_3_O_4_ induced a higher apoptosis rate of AdEPCs compared with CD133@Fe_3_O_4_ (Figure 5A,B). After incubating with 100 µg/mL PEG@Fe_3_O_4_ or CD133@Fe_3_O_4_ for 24 h, the OD values of AdEPCs–PEG@Fe_3_O_4_ and AdEPCs–CD133@Fe_3_O_4_ both decreased significantly (Figure 5C). Thus, based on the labeling efficiency and cytotoxicity of AdEPCs, we determined 50 µg/mL as the optimal labeling MNP concentration of AdEPCs magnetization and performed subsequent experiments with this concentration. Kawanishi M et al. proved that iron oxide nanoparticles usually induced more ROS production and subsequent oxidative stress-mediated responses [42]. Interestingly, we found that AdEPCs–CD133@Fe_3_O_4_ induced much less ROS production compared with AdEPCs–PEG@Fe_3_O_4_ at the concentration of 50 µg/mL (Figure 5D).

**Figure 6:**
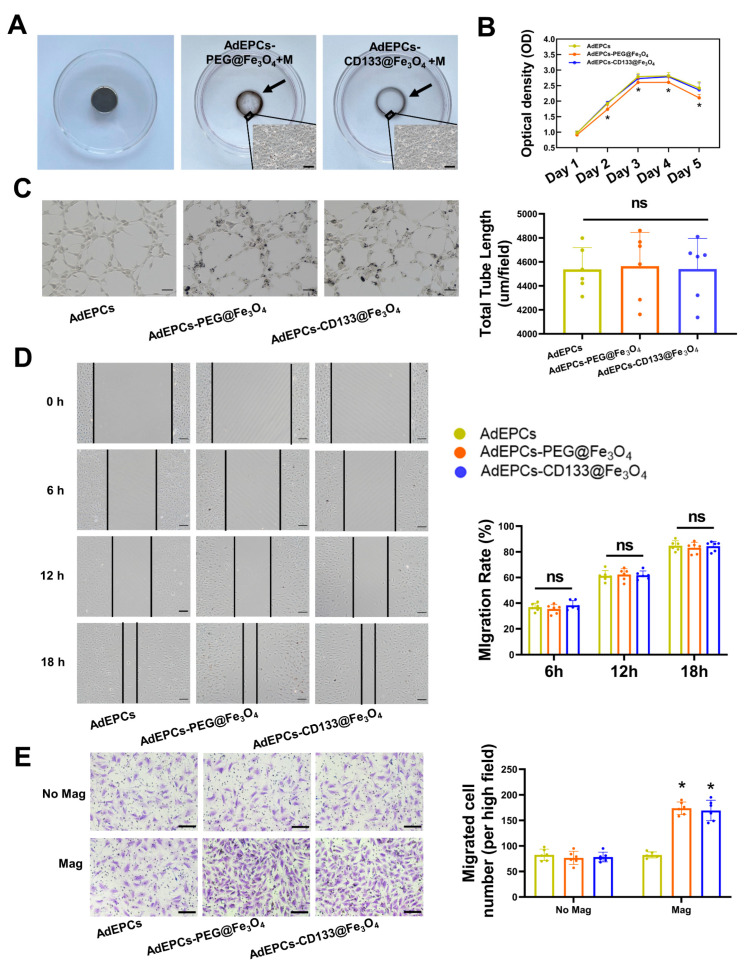
The differences between PEG@Fe_3_O_4_ and CD133@Fe_3_O_4_ on AdEPCs. (**A**) MNPs-labeled AdEPCs were seeded in separate cell culture dishes with a magnet placed underneath for 24 h. A cell circle could be observed (black arrow). Scale bar = 100 μm. (**B**) Proliferation curve of AdEPCs labeled with PEG@Fe_3_O_4_ and CD133@Fe_3_O_4_ for successive 5 days. * *p* < 0.05 vs. 0 μg/mL. (**C**) Representative images and statistical analysis of the tube formation capacities in the MNPs-labeled and unlabeled AdEPCs groups. Scale bar = 50 μm. (**D**) The scratch grooves of each group were detected at different time and quantified using the migration rate. (**E**) The transwell assays were performed to examine the effects of MNPs and magnetic guidance on the migration ability of AdEPCs. Mag/No Mag, with/without magnetic guidance. * *p* < 0.05 vs. AdEPCs with Mag group. Mag, magnetic guidance.

As shown in Figure 6A, after incubating AdEPCs–PEG@Fe_3_O_4_ and AdEPCs–CD133@Fe_3_O_4_ in separate cell culture dishes with a magnet on the lower surface for 24 h, two distinct cell circles could be observed. To investigate and compare the effects of PEG@Fe_3_O_4_ and CD133@Fe_3_O_4_ on the physiological functions of AdEPCs further, we performed CCK-8 assays, transwell assays, scratch assays, and tube formation assays. By measuring the OD values of cells for 5 consecutive days using CCK-8 assays, we found that the cell viability of AdEPCs–PEG@Fe_3_O_4_ decreased significantly after co-incubating for 2 days, while CD133@Fe_3_O_4_ maintained no significant effects on the cell viability of AdEPCs (Figure 6B). As shown in Figure 6C, both PEG@Fe_3_O_4_ and CD133@Fe_3_O_4_ did not influence the tube formation capability of AdEPCs by assessing the total tube lengths. Additionally, based on the scratch repair area of the three groups, no significant negative effects were observed between these two MNPs on the migration capacity of AdEPCs (Figure 6D). Moreover, in the presence of an external magnetic field, the number of migrated AdEPCs in transwell assays significantly increased in the magnetized groups compared with the no-magnetized groups (Figure 6E). In vitro results showed that magnetized AdEPCs could be distributed directionally under external magnetic guidance. Moreover, AdEPCs–PEG@Fe_3_O_4_ and AdEPCs–CD133@Fe_3_O_4_ shared similar migration and angiogenesis capacities, but CD133@Fe_3_O_4_ had fewer negative effects on cell proliferation and apoptosis of AdEPCs than PEG@Fe_3_O_4_.

### 3.5. In Vivo Tracking of AdEPCs

**Figure 7:**
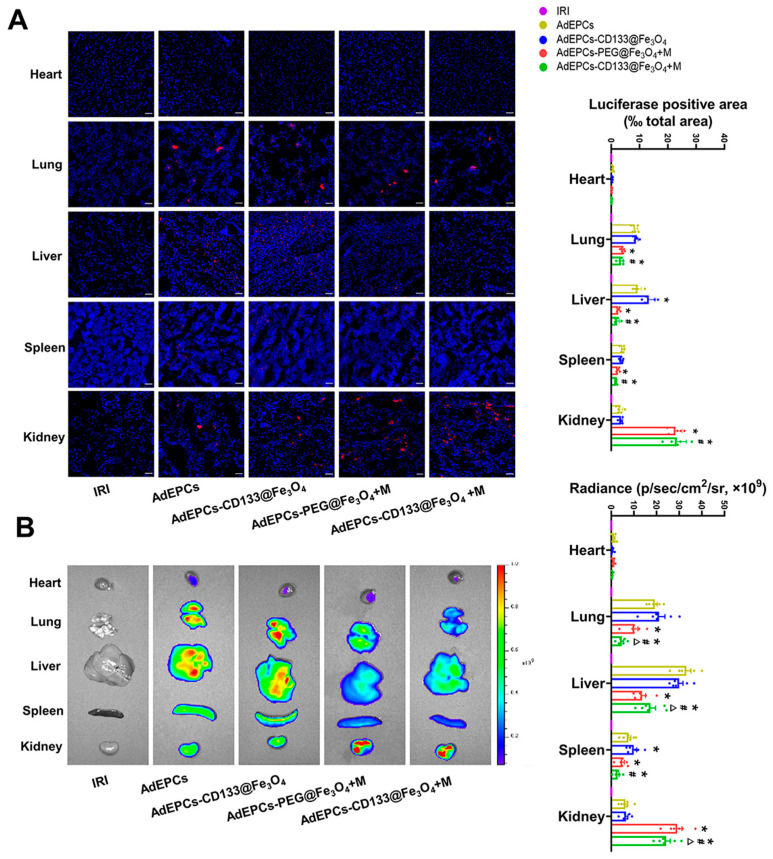
Representative fluorescence images and statistical analysis of major organs (heart, liver, spleen, lung, and kidney) of IRI rats detected by a fluorescent microscope (**A**) and in vivo imaging system (**B**). Scale bar = 50 μm. * *p* < 0.05 vs. AdEPCs group; # *p* < 0.05 vs. AdEPCs–CD133@Fe_3_O_4_ group; Δ *p* < 0.05 vs. AdEPCs–PEG@Fe_3_O_4_+M group.

After intravenous administration of CM-Dil and Dil-C18(5)-DS labeled AdEPCs with different treatments in vivo, we used fluorescence microscopy and an in vivo imaging system to track the distribution of AdEPCs. Semi-quantitative analysis of the fluorescence intensity of labeled AdEPCs suggested that the labeled AdEPCs could be detected in the liver, lung, and spleen, but few of them were observed in the heart. In addition, CD133@Fe_3_O_4_ would not affect the natural distribution of AdEPCs in the kidneys. However, when the renal magnetic guidance was exerted, both PEG@Fe_3_O_4_ and CD133@Fe_3_O_4_ could increase the AdEPCs retention in the injured kidney significantly, and no significant difference was found between them. Meanwhile, the retention of AdEPCs–CD133@Fe_3_O_4_ was significantly reduced in extrarenal organs after renal magnetic guidance (Figure 7A). The in vivo imaging revealed a similar trend except that the AdEPCs–PEG@Fe_3_O_4_+M group retained more AdEPCs in the kidneys than the AdEPCs–CD133@Fe_3_O_4_+M group (Figure 7B). Additionally, the in vivo tracking results of the AdEPCs–Iso@Fe_3_O_4_+M group and AdEPCs–CD133@Fe_3_O_4_+M group revealed that it was the CD133 that mediated the specific binding of AdEPCs to CD133@Fe_3_O_4_ rather than other components (Figures S1 and S2).

### 3.6. Outcomes of Renal Function and Tubular Damage

**Figure 8:**
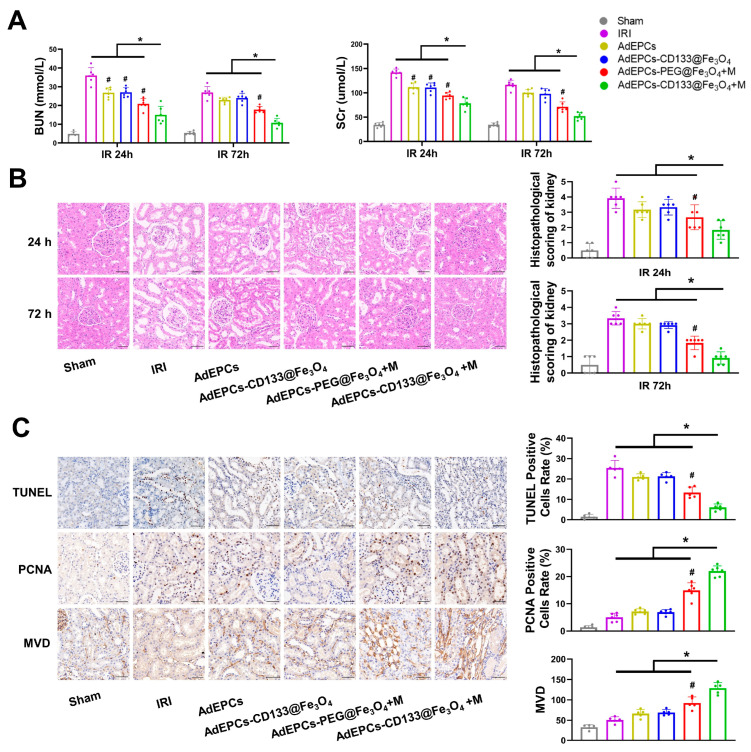
Effects of magnetically delivered AdEPCs on renal function. (**A**) Serum SCr and BUN in rats after 24 and 72 h of reperfusion. (**B**) Representative images of H&E staining and histopathological scoring of the kidneys at 24 and 72 h after reperfusion. (**C**) Representative images of TUNEL, PCNA, and MVD staining in the kidneys at 72 h after reperfusion. Scale bar = 50 μm. * *p* < 0.05 vs. AdEPCs–CD133@Fe_3_O_4_+M group; # *p* < 0.05 vs. IRI group.

As shown in Figure 8A, both BUN and SCr were significantly reduced in all AdEPCs transplantation groups after reperfusion at 24 h. Moreover, renal magnetic guidance of AdEPCs–PEG@Fe_3_O_4_ and AdEPCs–CD133@Fe_3_O_4_ could markedly rescue the renal function at both 24 and 72 h after renal IRI. The level of these renal injury markers in the AdEPCs–CD133@Fe_3_O_4_+M group was further reduced compared with all other five groups. Moreover, after 24 h of reperfusion, histological analyses of the kidney H&E staining sections revealed that AdEPCs transplantation had the trend of amelioration of tubular injury induced by IR, but this renoprotective effect was not significant compared with the IRI group. Instead, when IR kidney tissues were injected with magnetized AdEPCs and external renal magnetic guidance was exerted, we could observe an obvious reduction of tubular dilatation, necrosis, and vacuolization, whether 24 or 72 h after reperfusion. Notably, the renoprotection of the AdEPCs–CD133@Fe_3_O_4_+M group was even better than that of the AdEPCs–PEG@Fe_3_O_4_+M group at 24 and 72 h after reperfusion (Figure 8B). Although both PEG@Fe_3_O_4_ and CD133@Fe_3_O_4_ could significantly increase the number of AdEPCs in the injured kidneys under renal magnetic guidance, CD133@Fe_3_O_4_-treated AdEPCs have a superior restorative effect on renal IRI.

### 3.7. Cell Apoptosis and Proliferation, and Microvasculature in IRI Kidneys

To assess the potential renoprotection of AdEPCs with different MNPs on renal IRI further, we evaluated the proportion of TUNEL-positive and PCNA-positive renal cells as well as the MVD in kidney sections at 72 h after reperfusion (Figure 8C). Although a significant decrease of TUNEL-positive cells was observed in both the AdEPCs–CD133@Fe3O4+M group and the AdEPCs–PEG@Fe3O4+M group compared with the IRI group, the results showed a further decreased proportion of TUNEL-positive cells in the AdEPCs–CD133@Fe_3_O_4_+M group compared with other groups. The proportion of PCNA-positive cells also showed that magnetic delivery of AdEPCs could promote the proliferation of injured renal cells, while the AdEPCs–CD133@Fe_3_O_4_+M group had the most pronounced efficacy. As an indicator of MVD, CD34 was detected using immunohistochemistry. Evidence verified the benefits of magnetic delivery and that the increase of MVD was significantly higher in the AdEPCs–CD133@Fe_3_O_4_+M group than in the other groups. The AdEPCs–Iso@Fe_3_O_4_+M group served as an isotype control of the AdEPCs–CD133@Fe_3_O_4_+M group (Figures S3–S5). These results suggest that, in the renal IRI model, magnetic delivery of the magnetized AdEPCs could exert more significant renoprotective effects. Notably, compared with EM using PEG@Fe_3_O_4_, IM using CD133@Fe_3_O_4_ could further improve the therapeutic roles of AdEPCs on IRI kidneys.

### 3.8. The Effects of CD133^+^ AdEPCs on H/R HUVECs

**Figure 9:**
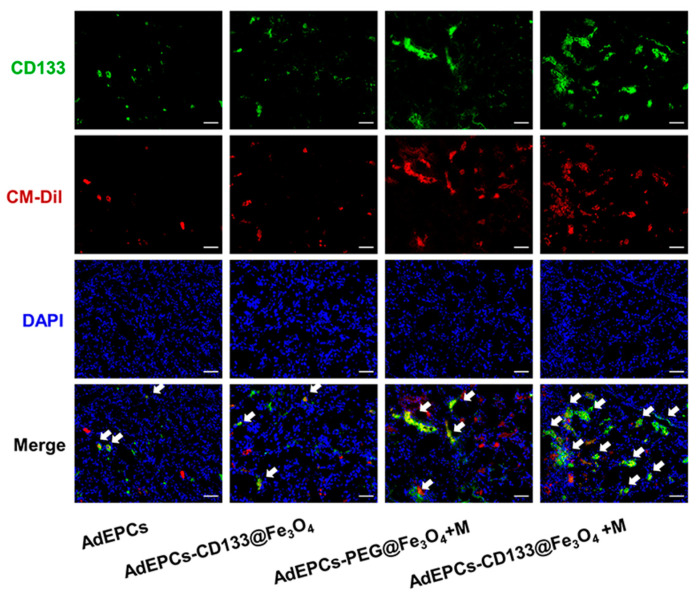
Representative images of CD133/CM-Dil double-positive AdEPCs (white arrow) in the four AdEPCs transplanted groups. Scale bar = 100 μm.

To investigate the reason for the stronger renoprotection of the AdEPCs–CD133@Fe_3_O_4_+M group, immunofluorescence staining of frozen kidney sections showed the presence of CD133^+^/CM-Dil^+^ cells in the four experimental groups. The AdEPCs–CD133@Fe_3_O_4_+M group revealed a significantly higher proportion of CD133^+^/CM-Dil^+^ cells than the other groups (Figure 9 and Figure S6). 

**Figure 10:**
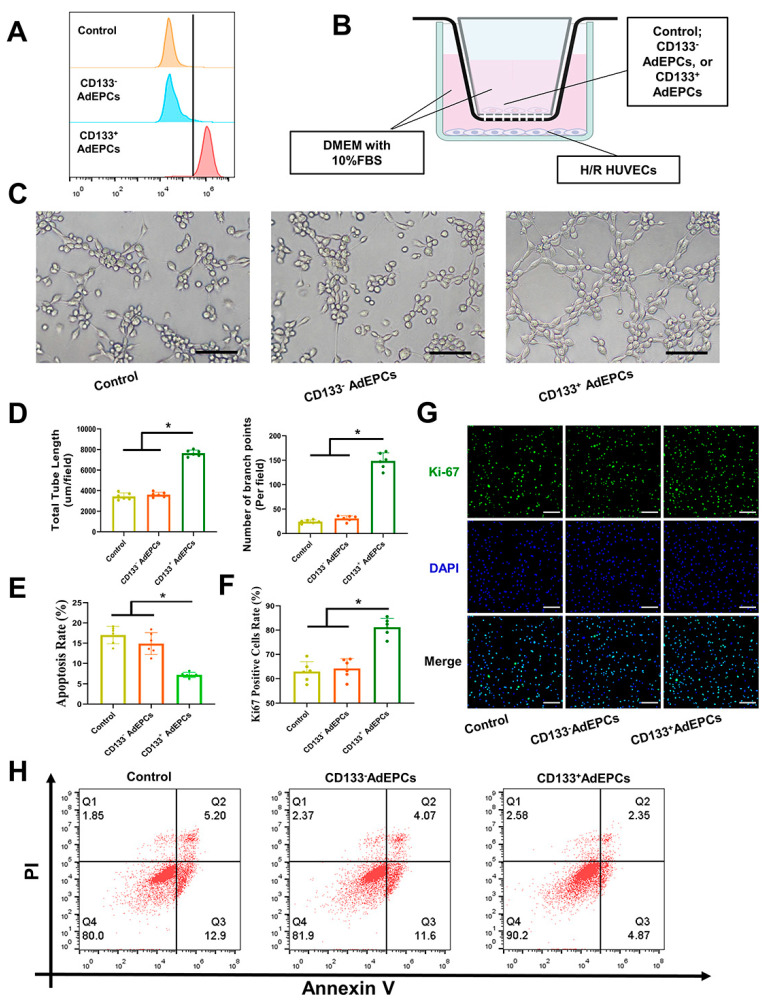
The effects of CD133^+^ AdEPCs on H/R HUVECs. (**A**) Flow cytometry assays of CD133 expression after magnetic cell sorting of AdEPCs–CD133@Fe_3_O_4_. (**B**) Experimental setup of AdEPCs–HUVECs 3D co-culture model. The lower chambers were seeded H/R-treated HUVECs with complete DMEM medium. The upper chambers contained complete DMEM alone, CD133^−^ AdEPCs, or CD133^+^ AdEPCs. Created with BioRender.com. (**C**,**D**) Representative images and quantified analysis of angiogenesis of H/R HUVECs after co-culture with medium alone, CD133^−^ AdEPCs, or CD133^+^ AdEPCs for 24 h. (**F**,**G**) Representative Ki-67 immunofluorescence staining images and Ki-67 positive cell rate of three groups. (**E**,**H**) The apoptosis rate of AdEPCs–HUVECs 3D co-culture models in three groups. Scale bar = 100 μm. * *p* < 0.05 vs. Control group.

After purifying the AdEPCs magnetized with CD133@Fe_3_O_4_ using the magnetic sorting column, we obtained CD133^+^ or CD133^−^ AdEPCs. Flow cytometry showed that the positive percentage of CD133^+^ AdEPCs collected was 90.2% ± 3.1% while the negative percentage was 4.42% ± 2.1% (Figure 10A). As shown in Figure 10B, using the AdEPCs–HUVECs 3D co-culture model, we found that CD133^+^ AdEPCs could significantly enhance the tube formation capability and cell proliferation of H/R HUVECs based on the results of total tube length, the number of branch points, and Ki-67 positive cells rate. Additionally, the apoptosis rate of the CD133^+^ AdEPCs group was also significantly decreased (Figure 10C–H).

## 4. Discussion

In this study, we first reported the IM of AdEPCs using CD133@Fe_3_O_4_ and investigated its feasibility for the non-invasive magnetic delivery of AdEPCs for the repair of renal IRI. Notably, we also constructed AdEPCs–PEG@Fe_3_O_4_ based on EM as a comparison. The current results demonstrate that both PEG@Fe_3_O_4_ and CD133@Fe_3_O_4_ could successfully magnetize AdEPCs. AdEPCs–PEG@Fe_3_O_4_ and AdEPCs–CD133@Fe_3_O_4_ had similar magnetic targeting abilities both in vitro and in vivo under magnetic guidance. Although the renoprotective effects of AdEPCs–PEG@Fe_3_O_4_ and AdEPCs–CD133@Fe_3_O_4_ were both enhanced under the renal magnetic guidance, AdEPCs–CD133@Fe_3_O_4_ had better therapeutic outcomes of renal function and tubular damage, probably due to its low cytotoxicity and its enrichment of a higher density of CD133^+^ AdEPCs in the injured kidneys. 

In recent years, researchers have reported the therapeutic roles of EPC transplantation derived from peripheral blood in renal IRI-induced AKI [13,14,43]. However, the renoprotection of AdEPCs has not been studied. As a subset of adipose-tissue-derived stem cells (ADSCs), AdEPCs possess ubiquitous abundance and could exert their therapeutic effects by differentiating into specific cell types and secreting cytokines in the injury sites [16,17,44]. The AdEPCs that we isolated and purified from rat autologous adipose tissues were found to express CD133, VEGFR2, CD31, and CD34 highly, with little expression of CD14 and CD45. Dil-Ac-LDL uptake and FITC-UEA-1 binding assays further confirmed our successful extraction and subculturing of AdEPCs. These results were consistent with previous studies on the definition of EPCs [9,17,45]. As previously mentioned, EPC transplantation has been widely demonstrated to provide renoprotection for AKI. However, only about 3% of the transplanted cells could successfully reach the kidney and become engrafted after intravenously applying EPCs without any other pretreatment [27]. Several studies used different pretreatments for EPCs to improve their renoprotective effects, but the low delivery efficiency of transplanted AdEPCs was inevitable [12,46,47,48]. 

Magnetic manipulation of EPCs with MNPs is a promising strategy that could significantly enhance the delivery efficiency in vivo. Based on EM or IM, both PEG@Fe_3_O_4_ and CD133@Fe_3_O_4_ fabricated in this study could magnetize AdEPCs. However, the current results show that PEG@Fe_3_O_4_ caused increased apoptosis, decreased proliferation, and increased ROS levels in AdEPCs. These cytotoxicities of PEG@Fe_3_O_4_ were stronger than those of CD133@Fe_3_O_4_ at the same incubation concentration. ROS-induced oxidative stress is one of the key toxicity mechanisms of nanomaterials. When MNPs enter the cytoplasm, the released free iron can react with hydrogen peroxide to produce free radicals [49]. Normally, cells can reduce ROS production effectively by upregulating the antioxidant defense. However, when the antioxidant cell-defense system is dysregulated, excessive ROS can cause cellular damage [50]. Although no further studies were conducted, we could speculate that the increased ROS formation of PEG@Fe_3_O_4_ in AdEPCs was the result of uncountable amounts of PEG@Fe_3_O_4_ that entered the cells through passive endocytosis.

By IM, the immune binding of CD133@Fe_3_O_4_ has significantly fewer negative effects on cellular physiological functions. Immunomagnetic bead purification is the most common cell sorting method and has been proven to have few effects on cell activity [51]. However, commercialized magnetic beads are often much larger than 200 nm, which is regarded as the largest size for biological applications [41]. Moreover, since its first discovery in 1997, CD133 has been widely studied as a stem cell and cancer stem cell marker. CD133 was thought to promote cell proliferation and angiogenesis and inhibit apoptosis, which is essential for the repair of renal IRI [19,52,53,54]. Meanwhile, CD133^+^ EPCs were proven to promote angiogenesis and have better therapeutic potential for vascular diseases [18,19,20,21,22,23,24]. Moreover, the isolated AdEPCs in this study highly expressed CD133. Therefore, we constructed CD133@Fe_3_O_4_ conjugated with CD133 antibody with the 20 nm Fe_3_O_4_ core for renoprotective applications. At the same incubation time, CD133@Fe_3_O_4_ could preferentially bind CD133^+^ AdEPCs, ensuring that CD133^+^ AdEPCs could complete magnetization. In contrast, PEG@Fe_3_O_4_ lacked this cell-specific magnetization. As shown in Figure 9, under the same renal magnetic guidance, the proportion of CD133^+^ AdEPCs in the AdEPCs–CD133@Fe_3_O_4_+M group was significantly higher than that in the AdEPCs–PEG@Fe_3_O_4_+M group.

The results of in vivo experiments showed a significant increase in AdEPCs retention in the injured kidneys and a decrease in the extrarenal organs after systematic transplantation of magnetized AdEPCs with renal magnetic guidance. Other analogous studies have similar trends [29,30,31,32]. Previous studies demonstrated that AdEPCs could be a promising angiogenic cell source for engineering bladder tissue [16]. In this study, transplantation of AdEPCs without magnetization had a trend of improving renal function and promoting repair of renal IRI, but the renoprotection of AdEPCs was not significant compared with the IRI group. However, magnetic delivery could significantly enhance the renoprotection of AdEPCs at 24 and 72 h after renal IRI. Interestingly, although the AdEPCs–PEG@Fe_3_O_4_+M group had a similar, or even higher, retention rate than the AdEPCs–CD133@Fe_3_O_4_+M group in injured kidneys, the latter had stronger renoprotective effects than the former. As mentioned above, this might be attributed to the surface-conjugated CD133 antibody of CD133@Fe_3_O_4_, which could introduce more CD133^+^ AdEPCs to the injured kidneys under renal magnetic guidance. To investigate further the therapeutic potential of CD133^+^ AdEPCs, CD133^+^ or ^−^ AdEPCs were co-cultured with H/R-treated HUVECs in vitro for 24 h, and the angiogenesis, cell proliferation, and apoptosis of H/R HUVECs in the CD133^+^ AdEPCs group were superior to those in the CD133^−^ AdEPCs and control groups. Besides being less cytotoxic to AdEPCs, these results suggest that the remarkable renoprotection of AdEPCs–CD133@Fe_3_O_4_ was, at least in part, due to the fact it could preferentially deliver large amounts of CD133^+^ AdEPCs to the injured kidney under magnetic guidance, while CD133^+^ AdEPCs could accelerate the recovery of renal IRI by repair of injured renal endothelial cells through paracrine mechanisms. Similarly, many studies have reported that EPCs could attenuate IRI or sepsis-induced AKI by releasing extracellular vesicles, which indicates the direction for further research in the future [10,55,56]. 

This study has some limitations. First, we did not explore the different therapeutic effects of diverse magnetic field strengths and time of duration to determine the optimal magnetic intensity for AdEPCs transplantation. Second, the long-term outcomes of transplanted AdEPCs and MNPs in renal IRI were not explored. Third, although AdEPCs were transplanted immediately after renal reperfusion in our study, this may be difficult to achieve in various clinical practices. The appropriate time of administration is worth studying further.

## 5. Conclusions

In this study, our results showed that magnetizing with CD133@Fe_3_O_4_ exerted minor negative effects on AdEPCs, and CD133@Fe_3_O_4_-mediated immunomagnetic delivery of AdEPCs could recruit more CD133^+^ AdEPCs to the injured kidneys and yield better renoprotection under renal magnetic guidance compared with magnetic delivery of AdEPCs using PEG@Fe_3_O_4_. Magnetizing AdEPCs with CD133@Fe_3_O_4_ could be a promising therapeutic strategy for renal IRI. Moreover, this approach can also be extended to other stem cells utilized for renoprotection.

## Data Availability

The datasets that support the findings of this study are available from the corresponding author upon reasonable request.

## Supplementary Materials

The following supporting information can be downloaded at: https://www.mdpi.com/article/10.3390/bioengineering10050509/s1, Figure S1: Analysis of cell distribution in tissue sections.; Figure S2: Analysis of cell distribution in solid organs.; Figure S3: Assessment of renal function.; Figure S4: Histopathological analysis of kidneys.; Figure S5: Renoprotection analysis.; Figure S6: Analysis of CD133^+^ AdEPCs distribution.
