# Supplementary material for: Immunomagnetic Delivery of Adipose-Derived Endothelial Progenitor Cells for the Repair of Renal Ischemia–Reperfusion Injury in a Rat Model
Source: Bioengineering (Basel). 2023 Apr 24;10(5):509. doi: 10.3390/bioengineering10050509 (PMC10215196; doi:10.3390/bioengineering10050509)
Supplement: Supplementary file 1 [file bioengineering-10-00509-s001.zip › bioengineering-2231499-supplementary.pdf]

Immunomagnetic delivery of adipose-derived endothelial progenitor cells for the repair of renal ischemia-reperfusion injury in a rat model

Di Wu [1,†], Jingyu Liu [1,†], Changcheng Zhou [1], Wenjie Ma [1], Liuhua Zhou [1], Yuzheng Ge [1] and Ruipeng Jia [1,*]

1 Department of Urology, Nanjing First Hospital, Nanjing Medical University, Nanjing, Jiangsu, People's Re-public of China;

† These authors contributed equally to this work.

* Correspondence: urojiarp@njmu.edu.cn.

Supplementary figures:

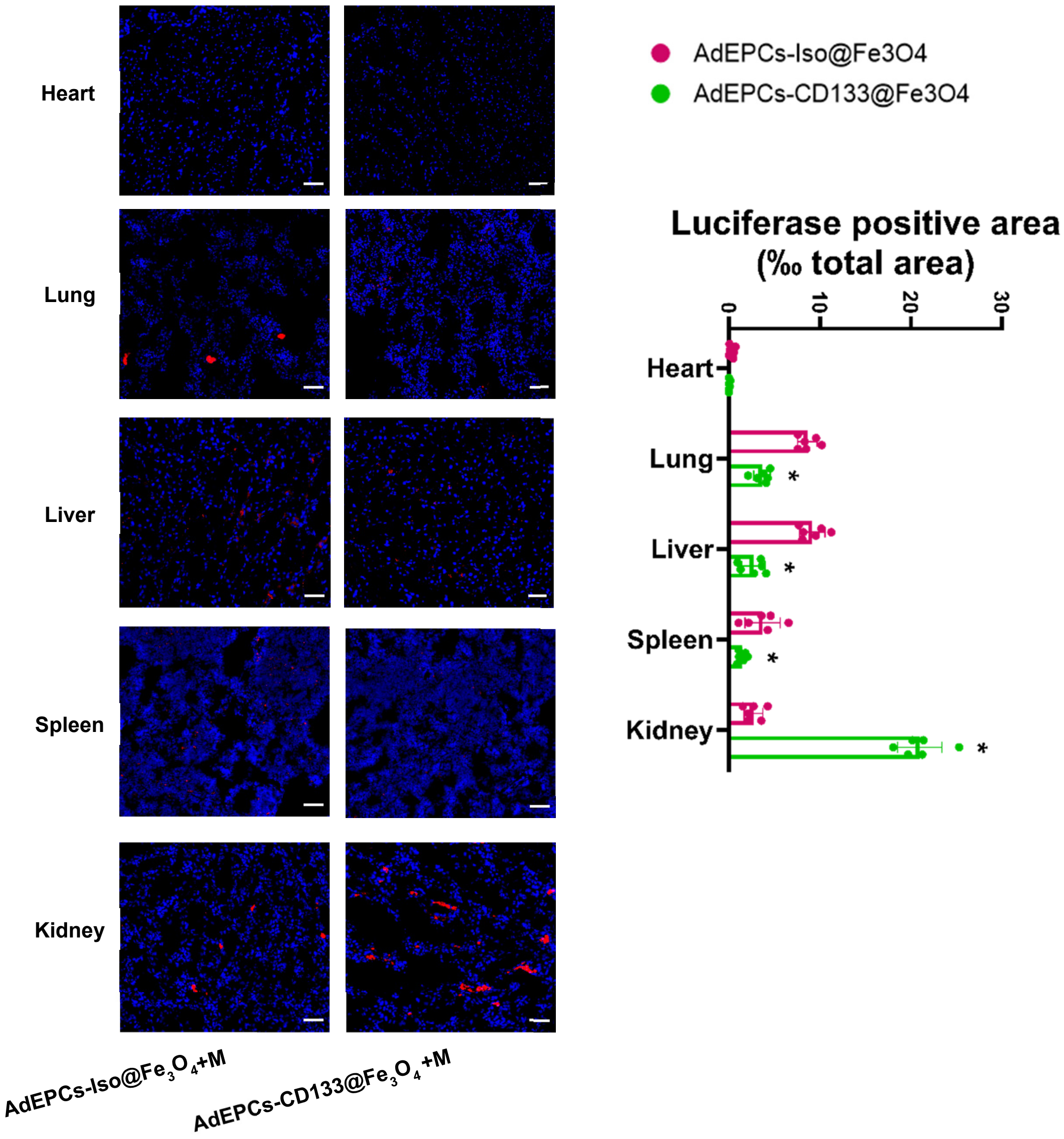

Figure S1. Analysis of cell distribution in tissue sections. Representative fluorescence images and statistical analysis of major organs (heart, liver, spleen, lung, kidney) of IRI rats detected by a fluorescent microscope. Scale bar=50 μm. * $p < 0.05$.

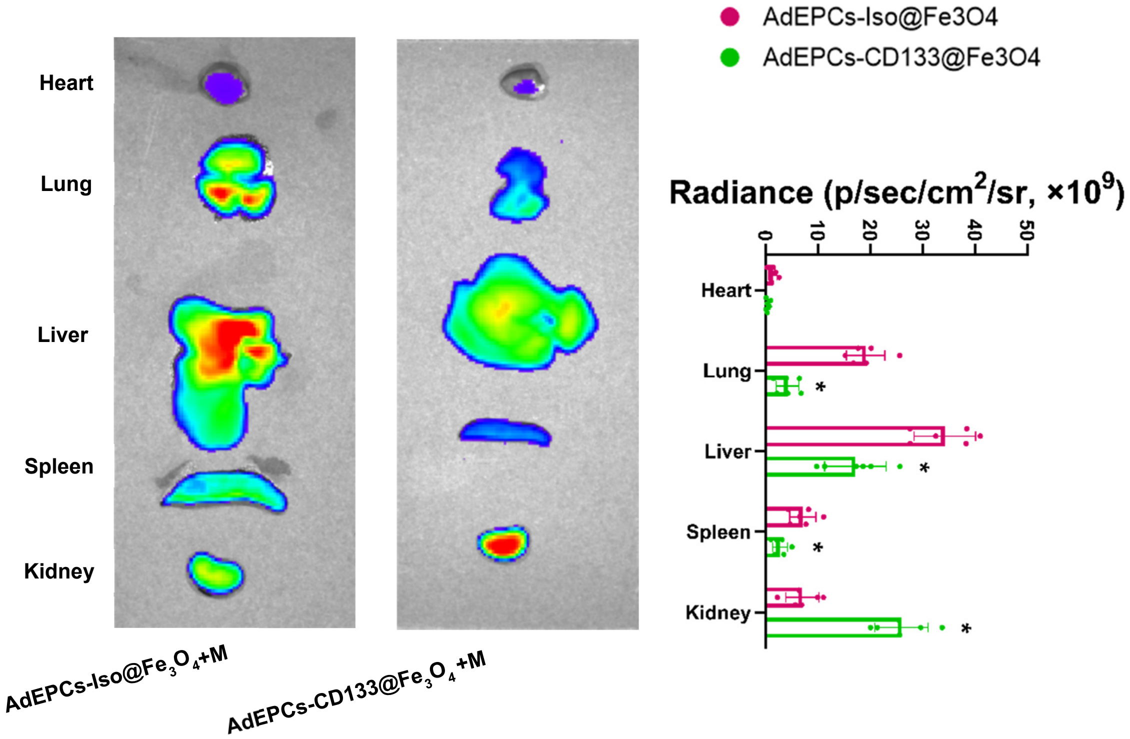

Figure S2. Analysis of cell distribution in solid organs. Representative images and statistical analysis of major organs (heart, liver, spleen, lung, kidney) of IRI rats detected by an IVIS Spectrum in vivo imaging system. * $p < 0.05$.

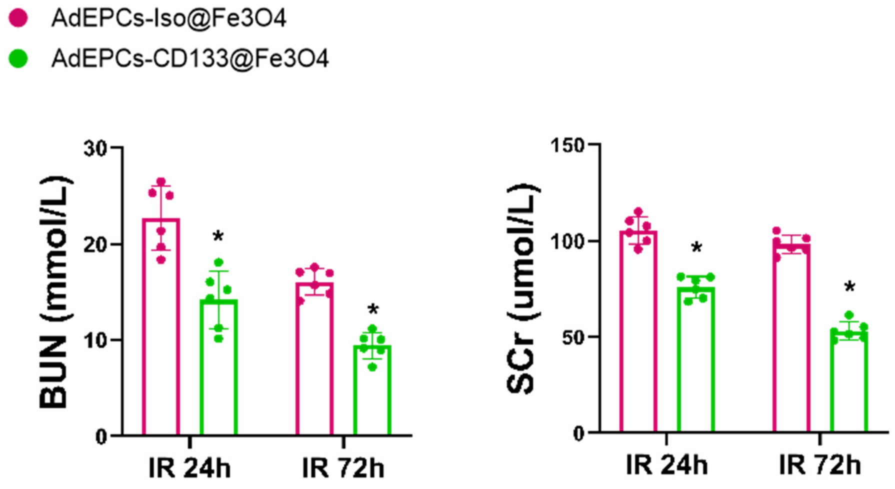

Figure S3. Assessment of renal function. The serum BUN and SCr of AdEPCs-Iso@Fe_3O_4+M group and AdEPCs-CD133@Fe_3O_4+M group after 24 and 72 h of reperfusion. * $p < 0.05$.

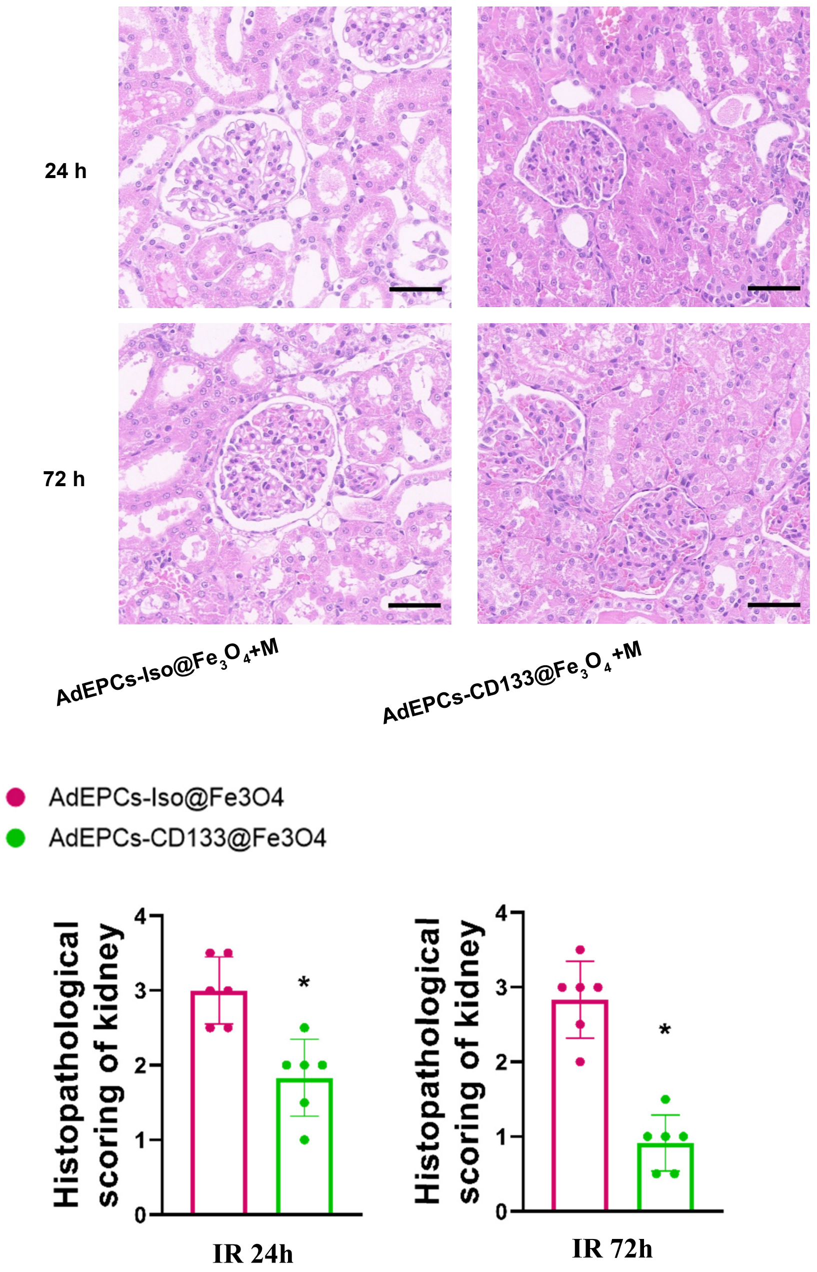

Figure S4. Histopathological analysis of kidneys. Representative images of H&E staining and histopathological scoring of the kidneys at 24 and 72 h after reperfusion. Scale bar=50 μm. * $p < 0.05$.

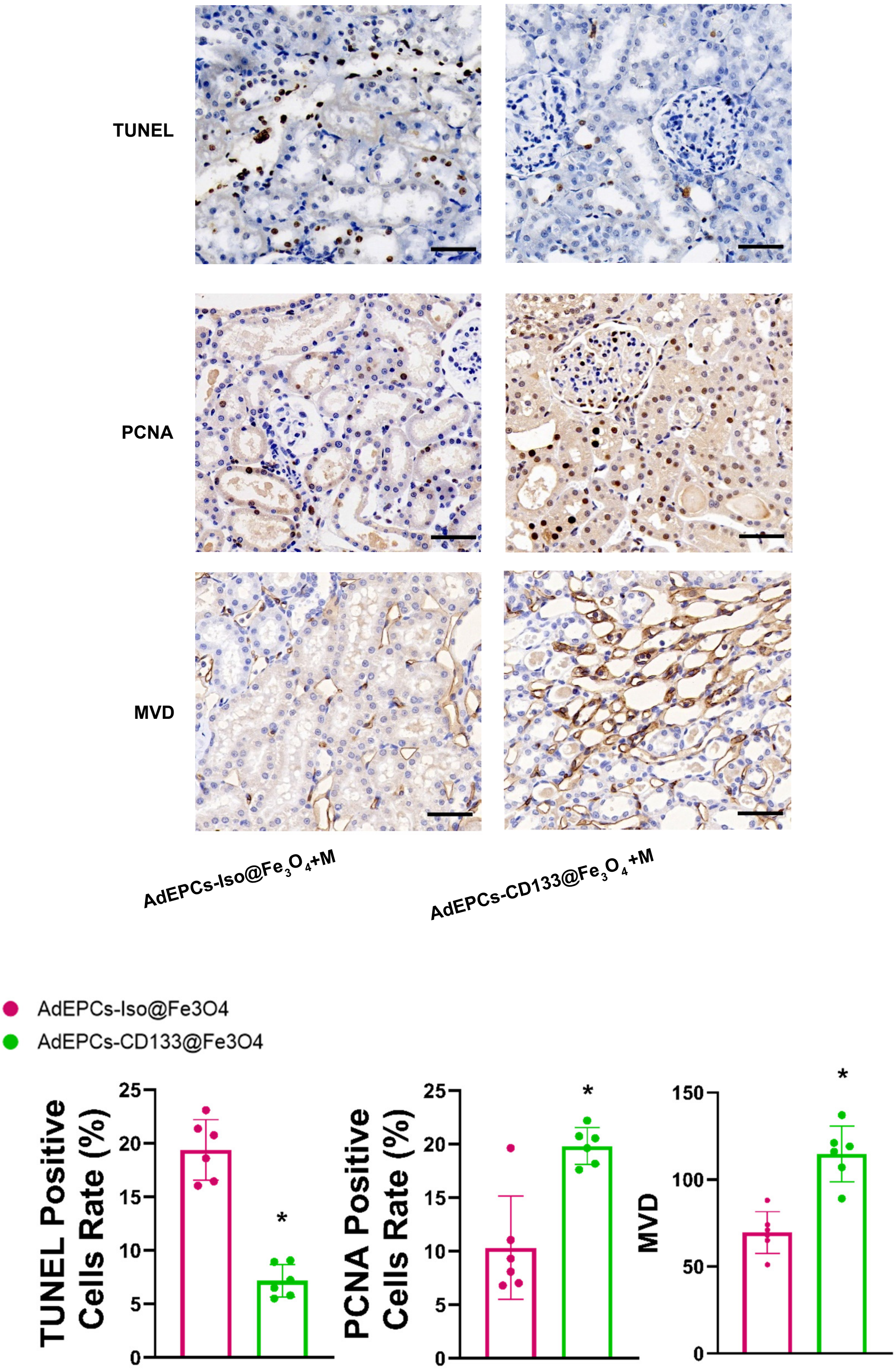

Figure S5. Renoprotection analysis. Representative images of TUNEL, PCNA, and MVD staining in the kidneys at 72 h after reperfusion. Scale bar=50 μm. * $p < 0.05$.

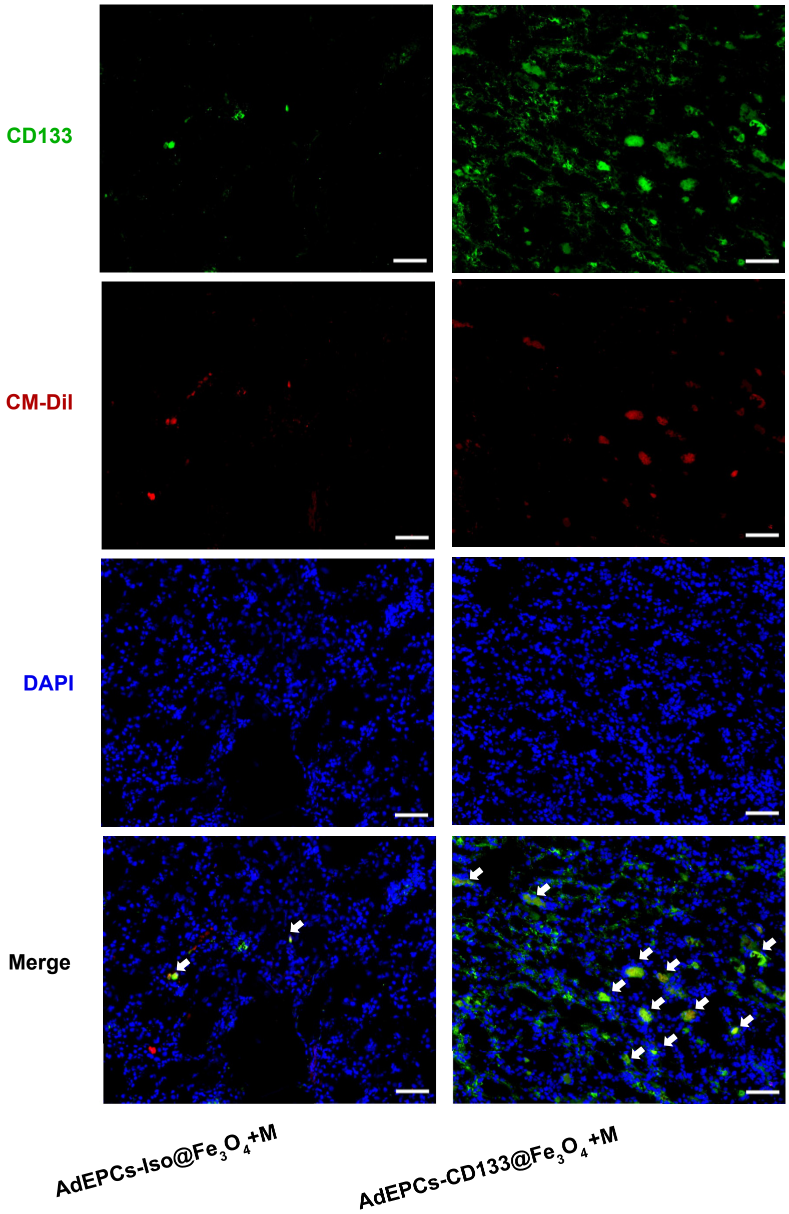

Figure S6. Analysis of CD133+ AdEPCs distribution. Representative images of CD133/CM-Dil double-positive AdEPCs (White arrows) in the AdEPCs-Iso@Fe_3O_4+M group and AdEPCs-CD133@Fe_3O_4+M group. Scale bar=100 μm. * $p < 0.05$.
